# Complete mitochondrail genome of *Corydoras agassizii*

**DOI:** 10.1080/23802359.2020.1715277

**Published:** 2020-01-20

**Authors:** Ligang Lv, Hang Su, Baohong Xu, Qiaoling Liu, Tiaoyi Xiao

**Affiliations:** a Hunan Engineering Technology Research Center of Featured Aquatic Resources Utilization, Hunan Agricultural University, Changsha, PR China;; b Collaborative Innovation Center for Efficient, Health Production of Fisheries in Hunan Province, Changde, PR China

**Keywords:** *Corydoras agassizii*, mitochondrail genome, next-generation sequencing, phylogenetic analysis

## Abstract

We reported the complete mitochondrial genome yielded using next-generation sequencing of *Corydoras agassizii* in this study. The total length of the mitochondrial genome is 16,562 bp, with the base composition of 32.6% A, 25.9% T, 26.8% C, and 14.7% G, in several. It contains two rRNA genes, 22 tRNA genes, 13 protein-coding genes, and a 945 bp non-coding control region (D-loop region). The sequence of these genes is consistent with that found in the Siluriformes. The complete mitogenomes of *C. agassizii* and other 17 species of fish were constructed by phylogenetic analysis using Neighbour-Joining method. The topological structure indicated that species participating in the analysis belong to three groups (Siluridae, Loricariidae, and Callichthyidae) of nine genera, and the *C. agassizi*i was clustered with other species from genus *Corydoras*. The external morphological characteristics of *C. agassizii* are consistent with the results of molecular classification, so the mitogenome can be used to identify *Corydoras* species in the future.

*Corydoras agassizii*, native to Amazon r. near Tabatinga, Brazil, belonging to Teleostei, Siluriformes, Callichthyidae, Corydoradinae, *Corydoras*, often called Agassiz’s Corydoras, the name is in honour of Dr. J. Louis r. Agassiz. *Corydoras agassizii* has soft rays from the first two or three backs that are black in length. Rays can vary in intensity and age.

In order to identify and distinguish the species more accurately, the mitochondrial genome sequence of *C. agassizii* was determined, and mitochondrial genome structure and phylogenetic analysis were performed. The living bodies of *C. agassizii* were collected from the Red Star Ornamental Fish Market in Changsha, Hunan Province, China (113.03 E, 28.09 N). The fish bodies were anesthetized with MS-222 (3-Aminobenzoic acid ethyl ester methanesulfonate) and the dorsal muscle tissues were collected and preserved in 99% ethanol in Museum of Hunan Agricultural University. After extracting the DNA of the tissue (Number: AKX002), the sequence library was constructed. The terminal readings were paired with HiSeq XTen PE 150 of Illumina. BBduk and BLAST+ were used to evaluate and monitor data quality. NOVOPlasty and SPAdes were used for ab initio assembly of mitochondrial genome (Liu et al. 2019; Tan et al. 2019). MITOS2 server and Geneious R11 were used to predict and annotate mitochondrial genome. Geneious Tree Builder was used for phylogenetic analysis and construction of phylogenetic tree (Liu et al. 2019).

A total of 21,595,706 high-quality clean readings (150 bp PE reading length) were obtained in the analysis. The total length of the mitochondrial genome is 16,562 bp (GenBank accession number: MN641875), with the base composition of 32.6% A, 25.9% T, 26.8% C, and 14.7% G, in several. It contains two rRNA genes, 22 tRNA genes, 13 protein-coding genes, and a short non-coding control region 945 bp region (D-loop region). The sequence of these genes is consistent with that found in the Siluriformes (Saitoh et al. 2003). Except for the protein COX1, which begins with TGT, all other protein initiation codons begin with ATG. The complete mitogenomes of *C. agassizii* and other 17 species of fish were constructed by phylogenetic analysis using Neighbour-Joining method. The topological structure indicated that species participating in the analysis belong to three groups (*Siluridae*, *Callichthyidae*, and *Loricariidae*) of nine genera, and the *C. agassizi*i was clustered with other species from genus *Corydoras* (Figure 1). The external morphological characteristics of *C. agassizii* are consistent with the results of molecular classification, so the mitogenome can be used to identify *Corydoras* species in the future (Betancur-R et al. 2017).

**Figure 1.**
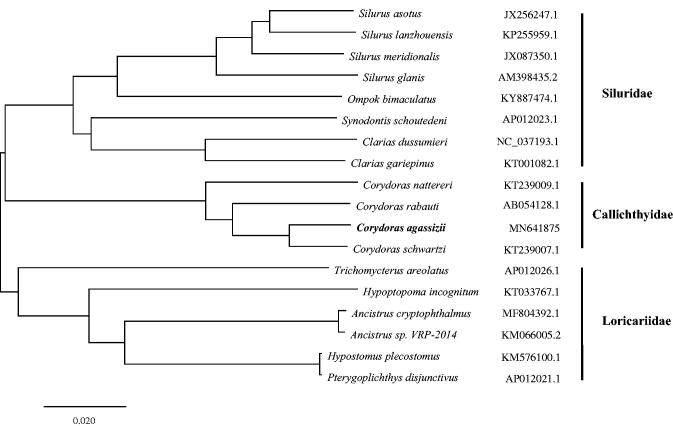
Neighbour-Joining phylogenetic tree based on the complete mitochondrial genome sequence. *Note*: the bold Latin name represents the species in this study. The codes followed the Latin names were GenBank accession numbers for each mitogenomes.
